# Supplementary material for: Cul3 and insomniac are required for rapid ubiquitination of postsynaptic targets and retrograde homeostatic signaling
Source: Nat Commun. 2019 Jul 5;10:2998. doi: 10.1038/s41467-019-10992-6 (PMC6611771; doi:10.1038/s41467-019-10992-6)
Supplement: Supplementary file 1 — Supplementary Information [file 41467_2019_10992_MOESM1_ESM.pdf]

Supplementary Information

insomniac and *Cul3* are required for rapid ubiquitination of postsynaptic targets and retrograde homeostatic signaling

Kikuma et al.

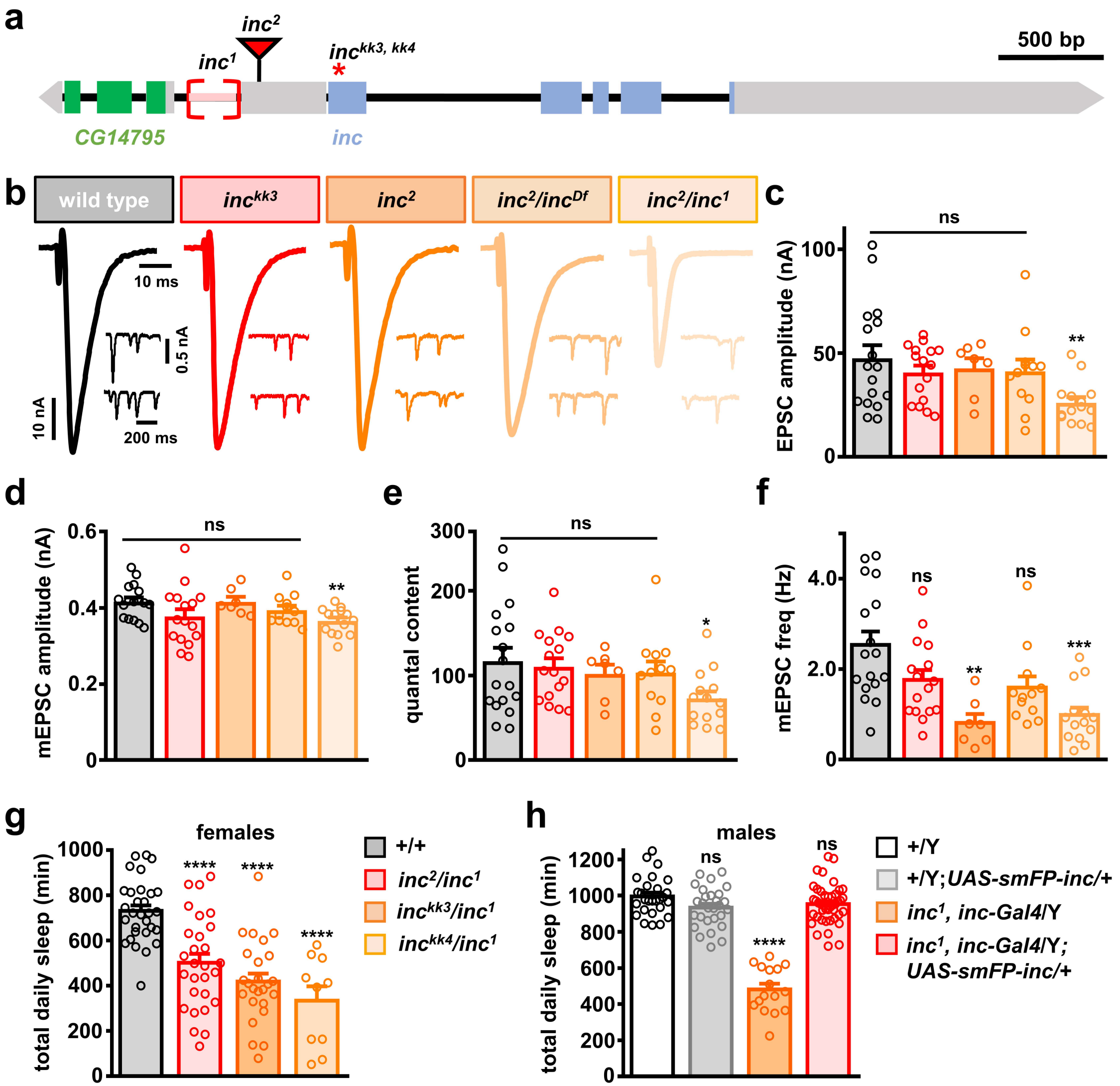

a
inc2
inckk3, kk4
inc1
500 bp
CG14795
inc
b
wild type
inckk3
inc2
inc2/incDf
inc2/inc1
10 ms
0.5 nA
10 nA
200 ms
c
ns
**
EPSC amplitude (nA)
d
ns
**
mEPSC amplitude (nA)
e
ns
*
quantal content
f
ns
**
ns
***
mEPSC freq (Hz)
g
females
****
****
****
total daily sleep (min)
+/+
inc2/inc1
inckk3/inc1
inckk4/inc1
h
males
ns
****
ns
total daily sleep (min)
+/Y
+/Y;UAS-smFP-inc/+
inc1, inc-Gal4/Y
inc1, inc-Gal4/Y;
UAS-smFP-inc/+

Supplementary Figure 1: *inc* mutants generated by CRISPR/Cas9 gene editing exhibit normal baseline transmission and the expected defects in sleep behavior. **(a)** Schematic of the *Drosophila inc* locus. The deleted region of inc^1, the pBac transposon insertion site of inc^2, and the CRISPR-induced indel mutations in inc^{kk3} and inc^{kk4} (*) are shown. **(b)** Representative electrophysiological traces of EPSCs and mEPSCs for wild type (w^{1118}), inc^{kk3}, inc^2, inc^2/inc^{Df}, and inc^2/inc^1 mutants. While inc^2/inc^1 mutants show reduced synaptic transmission, baseline synaptic transmission is largely normal in the other *inc* alleles. Quantification of average EPSC amplitude **(c)**, mEPSC amplitude **(d)**, quantal content **(e)**, and mEPSC frequency **(f)** values in wild type (w^{1118}, n=17), inc^{kk3} (n=16), inc^2 (n=7), inc^2/inc^{Df} (n=12), and inc^2/inc^1 (n=14) mutants. **(g)** Quantification of average daily sleep in female flies of the indicated genotype. inc^{kk3}/inc^1 and inc^{kk4}/inc^1 females show reduced daily sleep, similar to inc^1/inc^2 transhetrozygotes (wild type, n=30; inc^2/inc^1, n=28; inc^{kk3}/inc^1, n=25; inc^{kk4}/inc^1, n=10). **(h)** Expression of *UAS-smFP-inc* driven by *inc-Gal4* restores sleep to wild-type levels in male inc^1 mutants (wild type, n=27; *UAS-smFP-inc/*+, n=28; inc^1*,inc-Gal4/Y*, n=16; inc^1*,inc-Gal4/Y;UAS-smFP-inc/*+*,* n=43). Asterisks indicate statistical significance using a one-way ANOVA followed by Tukey’s multiple comparison test: (*) p<0.05; (**) p<0.01; (***) p<0.001; (****) p<0.0001, (ns) not significant. Error bars indicate ±SEM. n values indicate biologically independent cells (c-f), or biologically independent animals for sleep behavior (g-h). Additional statistical information can be found in Supplementary Table 3. Source data are provided in the Source Data file.

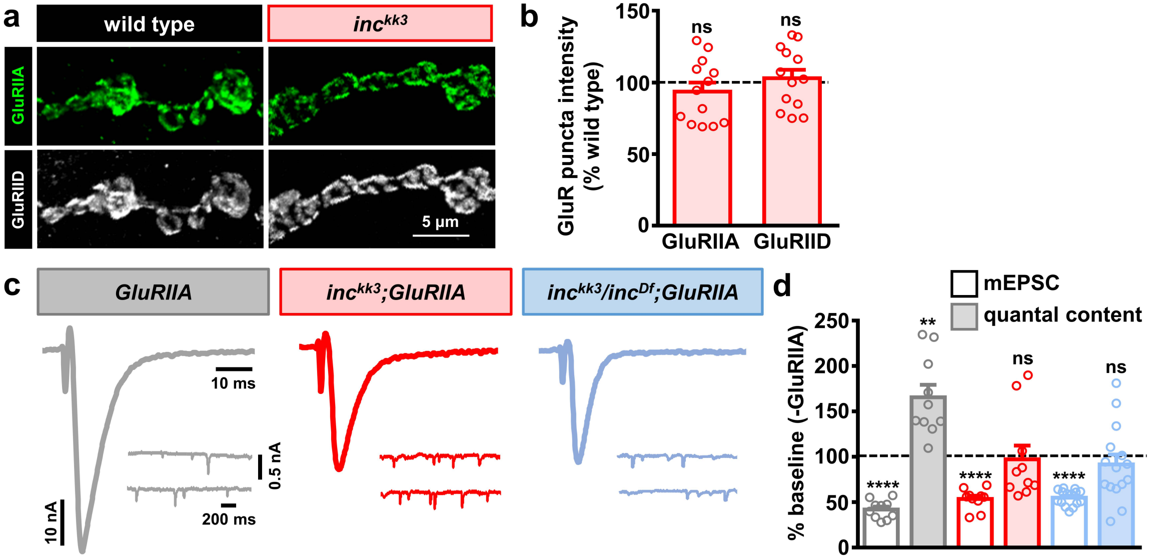
a
wild type
inc^kk3
GluRIIA
GluRIID
5 µm
b
GluR puncta intensity (% wild type)
150
100
50
0
ns
ns
GluRIIA
GluRIID
c
GluRIIA
inc^kk3;GluRIIA
inc^kk3/inc^Df;GluRIIA
10 ms
0.5 nA
200 ms
10 nA
d
mEPSC
quantal content
% baseline (-GluRIIA)
250
200
150
100
50
0
****
**
****
ns
****
ns

Supplementary Figure 2: *inc* is required for the chronic expression of PHP. (a) Representative images from wild type and inc^{kk3} mutant NMJs immunostained with antibodies against the postsynaptic glutamate receptor subunits GluRIIA and GluRIID. No alteration in glutamate receptor levels is observed in *inc* mutants. **(b)** Quantification of mean fluorescence intensity levels of GluRIIA and GluRIID (wild type, n=10; inc^{kk3}, n=13). **(c)** Representative EPSC and mEPSC traces from the indicated genotypes. PHP fails to be expressed in inc^{kk3} mutants when combined with *GluRIIA* mutants (inc^{kk3};$GluRIIA^{SP16}$). **(d)** Quantification of mEPSC and quantal content values in *GluRIIA* (n=10), inc^{kk3};*GluRIIA* (n=10), inc^{kk3}/inc^{Df};*GluRIIA* (n=15) normalized to baseline conditions (-*GluRIIA*). Asterisks indicate statistical significance using a Student's t test: (**) p<0.01; (****) p<0.0001, (ns) not significant. Error bars indicate ±SEM. n values indicate biologically independent cells. Additional statistical information and absolute values for normalized data can be found in Supplementary Table 3. Source data are provided in the Source Data file.

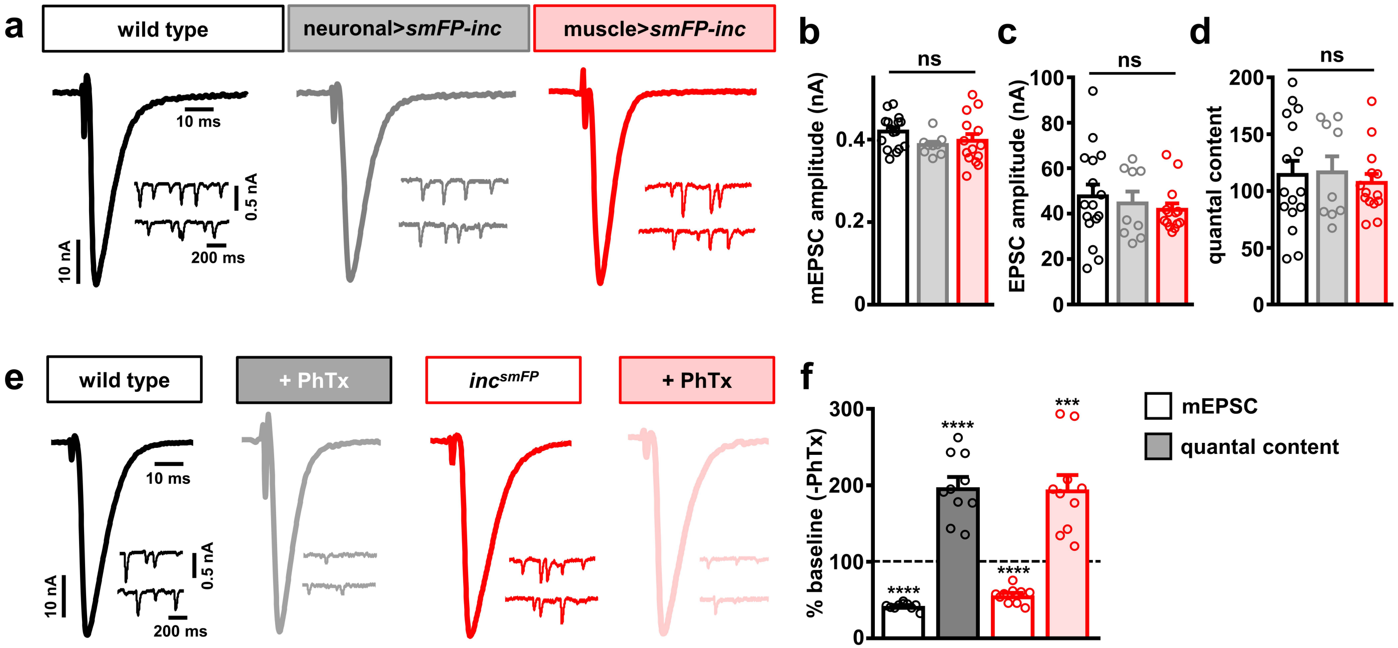
a
wild type
neuronal>smFP-inc
muscle>smFP-inc
10 ms
0.5 nA
200 ms
10 nA
b
ns
mEPSC amplitude (nA)
0.4
0.2
0
c
ns
EPSC amplitude (nA)
100
80
60
40
20
0
d
ns
quantal content
200
150
100
50
0
e
wild type
+ PhTx
inc^smFP
+ PhTx
10 ms
0.5 nA
10 nA
200 ms
f
% baseline (-PhTx)
300
200
100
0
****
****
****
***
mEPSC
quantal content

Supplementary Figure 3: Overexpression and endogenous tagging of *inc* does not perturb baseline neurotransmission or PHP expression. (a) Representative EPSC and mEPSC traces of wild type, neuronal *inc* overexpression (neuronal>*smFP-inc*: *OK371-Gal4/UAS-smFP-inc*), and muscle *inc* overexpression (muscle>*smFP-inc*: *UAS-smFP-inc/+;MHC-Gal4/+*). Quantification of mEPSC amplitude **(b)**, EPSC amplitude **(c)**, and quantal content **(d)** values from wild type (n=16), neuronal>*smFP-inc* (n=9), and muscle>*smFP-inc* (n=14). PHP is expressed in these genotypes after PhTx application (Supplementary Table 3). **(e)** Representative EPSC and mEPSC traces from wild type and endogenously tagged inc^{smFP} before and after PhTx application. Synaptic transmission and PHP function similarly to wild type in inc^{smFP}. **(f)** Quantification of average mEPSC amplitude and quantal content values following PhTx application relative to baseline (-PhTx) (wild type baseline, n=10; inc^{smFP} baseline n=11; wild type + PhTx, n=10; inc^{smFP}+ PhTx, n=10). Asterisks indicate statistical significance using a one-way ANOVA followed by Tukey's multiple comparison test (b-d) or a Student's t test (f): (***) p<0.001; (****) p<0.0001, (ns) not significant. Error bars indicate ±SEM. n values indicate biologically independent cells. Additional statistical information and absolute values for normalized data can be found in Supplementary Table 3. Source data are provided in the Source Data file.

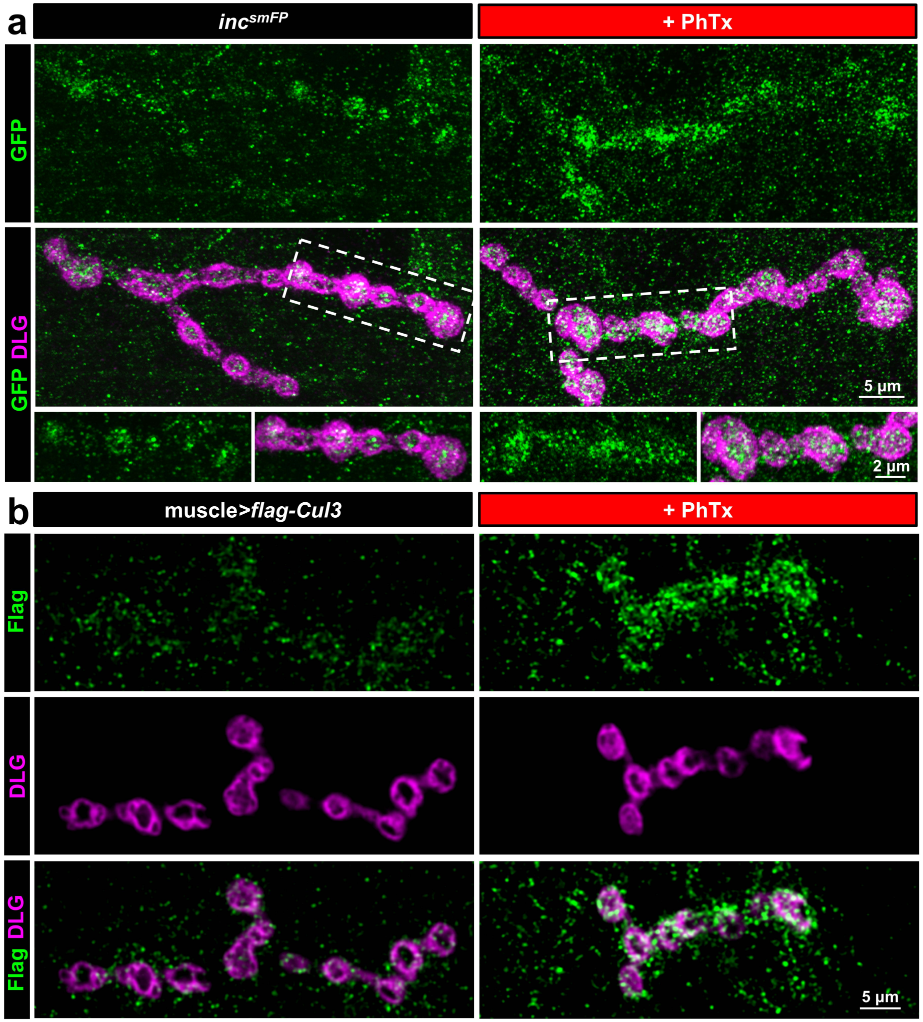

a
inc^smFP
+ PhTx
GFP
GFP DLG
5 µm
2 µm
b
muscle>flag-Cul3
+ PhTx
Flag
DLG
Flag DLG
5 µm

Supplementary Figure 4: Inc^{smFP} and Flag-Cul3 rapidly accumulate at the NMJ following PhTx application. Representative images of the muscle 6 NMJ in *inc^{smFP}* larvae **(a)** and *flag-Cul3* expressed in the postsynaptic muscle (*G14-Gal4/UAS-3xHA-3xflag-Cul3*) **(b)** at baseline and after 10 min incubation in PhTx. NMJs are immunostained with anti-GFP or anti-Flag and anti-DLG.

Supplementary Table 1: List of Inc- and Cul3-interacting genes screened and summarized results. The gene identity, name, putative function, genotype, genetic perturbation, source, and mEPSP, EPSP, and quantal content values are shown for each gene screened. The reference indicates the source that reported an interaction between Inc or Cul3 or their mammalian homologs.

CG #	Gene	Abb.	Putative function	Allele	Disruption Type	Source	PhTx	mEPSP	EPSP	QC	n	Ref.
**Inc- interacting genes**												
			**w1118**				+	0.6	33	63.9	7	
							-	1.1	35	32.4	12	
9025	Fem-1	Fem-1	regulate Stem-Loop Binding Protein (SLBP) translation and degradation	Fem-1^{EP2065}	transposon insertion in 5' UTR	BL17232	+	0.6	32	53.9	7	Giot et al., 2003[2]
							-	1.1	28	27.6	8	
7001	S6 Kinase Like	S6KL	inhibits BMP signaling, neuromuscular junction growth and synaptic endocytosis	S6KL^{EY06723}	transposon insertion in 5' UTR	BL16752	+	0.6	29	50.0	8	Giot et al., 2003[2]
							-	1.1	30	30.2	8	
3937	cheerio	cher	neuronal growth cones, dimeric F-actin crosslinking protein	cher^{MI07480}	transposon insertion in intron	BL43714	+	0.6	30	51.1	5	Giot et al., 2003[2]
							-	1.0	33	35.7	6	
8593	quemao	qm	catalyses protein prenylation and facilitates membrane targeting of proteins	qm^{2}	transposon insertion in 3' UTR	BL25827	+	0.5	19	34.2	7	Giot et al., 2003[2]
							-	1.1	23	22.4	7	
7809	Grasp65	Grasp65	myristoylated Golgi protein, mediate the Golgi bypass of transmembrane proteins	Grasp^{302}	transposon insertion in 5' UTR	Zhou et al., 2014	+	0.6	32	57.7	12	Giot et al., 2003[2]
							-	1.3	31	26.2	14	
7809	Grasp65	Grasp65	myristoylated Golgi protein, mediate the Golgi bypass of transmembrane proteins	Grasp^{129}	transposon insertion in 5' UTR	Zhou et al., 2014	+	0.6	29	53.4	14	Giot et al., 2003[2]
							-	1.2	38	37.5	8	
6606	Rab11 interacting protein	Rip11	binds to GTP bound form of Rab11, regulates Rhodopsin transport and cytokinesis	Rip11^{KG02485}/+	transposon insertion in 5' UTR	BL13742	+	0.5	30	62.5	4	Giot et al., 2003[2]
							-	0.7	34	51.2	5	
4593	Sex lethal	Sxl	RNA-binding, switch sexual dimorphism	Sxl^{f2}	spontaneous	BL54593	+	0.7	32	40.6	4	Giot et al., 2003[2]
							-	1.2	33	28.2	4	
3018	lesswright	lwr	innate immunity, meiosis, and anterior patterning of the embryo	lwr^{05486}/+	transposon insertion in exon	BL11410	+	0.5	33	73.4	2	Giot et al., 2003[2]
							-	0.9	35	40.1	3	
14899	Derlin-2	Der-2	regulate ER degradation	Der-2^{HP30303}	transposon insertion in 5' UTR	BL22205	+	0.6	35	54.5	5	Hein et al., 2015[3]
							-	1.1	37	32.6	8	
32654	Secretory 16	Sec16	organize endoplasmic reticulum exit sites, stress response	Sec16^{A}	ethyl methanesulfonate	BL52390	+	0.7	36	49.7	11	Hein et al., 2015[3]
							-	1.0	36	38.2	7	
11642	TRAM	TRAM	pain sensitive, immune responses	TRAM^{KG01407}	transposon insertion in 5' UTR	BL13435	+	0.5	30	60.2	4	Hein et al., 2015[3]
							-	1.5	37	24.4	4	
8783	Cdc42	Cdc42	GTPase signaling protein, regulate actin cytoskeleton	Cdc42^{1}	ethyl methanesulfonate	BL8783	+	0.4	41	101.1	2	Hein et al., 2015[3]
							-	1.0	42	38.0	4	
**CG #**	**Gene**	**Abb.**	**Putative function**	**RNAi**	**Genotype**	**Source**	**PhTx**	**mEPSP**	**EPSP**	**QC**	**n**	**Ref.**
7937	C15	C15	Unknown	TRiP 20	G14-Gal4X TRiP.HMS01431	BL35018	+	0.7	34	52.3	7	Giot et al., 2003[2]
							-	1.4	39	27.9	2	
6223	Coat Protein (coatomer) β	βCOP	retrograde transport of vesicles from the golgi to the endoplasmic reticulum	TRiP 20	G14-Gal4X TRiP.HMS01079	BL33741	+	0.6	31	52.4	8	Hein et al., 2015[3]
							-	0.9	32	34.7	8	
16728	G protein-coupled receptor kinase interacting ArfGAP	Git	muscle morphogenesis, synaptic vesicle recycling	TRiP 10	G14-Gal4X TRiP.JF01156	BL31583	+	0.5	30	60.1	6	Wang et al., 2011[7]
							-	0.9	37	41.9	6	
33052	Golgin, RAB6 interacting	Gorab	locomotion, cell cycle, spermatogenesis	TRiP 20	G14-Gal4X TRiP.HMC03748	BL55610	+	0.7	36	55.3	7	Hein et al., 2015[3]
							-	1.2	35	32.1	7	
7359	Secretory 22	Sec22	regualte Golgi apparatus, ER	TRiP 20	G14-Gal4X TRiP.HMS01238	BL34893	+	0.4	30	80.2	4	Hein et al., 2015[3]
							-	0.8	30	39.5	3	
9206	Dynactin 1, p150 subunit	DCTN1-p150	modulates binding of dynein to cellular cargoes, neuronal transport and neurogenesis	dominant negative	G14-Gal4X UAS-DCTN1-p150^{A}	BL51645	+	0.5	32	63.3	3	Hein et al., 2015[3]
							-	1.0	34	35.2	6	
5000	mini spindles	msps	microtubule dynamic, oogenesis	TRiP 10	G14-Gal4X TRiP.JF01613	BL31138	+	0.6	43	77.9	4	Hutchins et al., 2010[5]
							-	1.1	40	37.5	4	
**Cul3- interaction genes**												
**CG #**	**Gene**	**Abb.**	**Putative function**	**Allele**	**Disruption Type**	**Source**	**PhTx**	**mEPSP**	**EPSP**	**QC**	**n**	**Ref.**
9148	supercoiling factor	scf	endoplasmic reticulum-resident protein	scf^{EY01124}	transposon insertion in 5' UTR	BL15325	+	0.5	28	54.3	8	Bennett et al., 2010[1]
							-	1.0	29	28.2	20	
17765	peflin	PEF1	ER-to-Golgi transport, calcium-dependent adaptor, collagen export	CG17765^{C295}	transposon insertion in intron	BL16330	+	0.6	12	22.3	15	McGourty et al., 2016[6]
							-	1.1	32	29.8	15	
7210	kelch	kel	ubiquitin-proteasome pathway	kel^{DE1}	ethyl methanesulfonate	BL4893	+	0.6	35	60.0	10	Hudson et al., 2015[4]
							-	1.1	34	31.4	6	
1250	Secretory 23	Sec23	Golgi, ER membrane transport regulation	Sec23^{EY06757}	transposon insertion in 5' UTR	BL15325	+	0.4	23	52.1	3	McGourty et al., 2016[6]
							-	1.2	32	30.0	3	
**CG #**	**Gene**	**Abb.**	**Putative function**	**RNAi**	**Genotype**	**Source**	**PhTx**	**mEPSP**	**EPSP**	**QC**	**n**	**Ref.**
7210	kelch	kel	ubiquitin-proteasome pathway	TRiP 10	G14-Gal4X	BL31251	+	0.5	34	65.0	8	Hudson et al., 2015[4]
							-	1.1	33	33.4	8	
9148	supercoiling factor	scf	endoplasmic reticulum-resident protein	TRiP 20	G14-Gal4X	BL34331	+	0.6	28	48.9	6	Giot et al., 2003[2]
							-	0.9	32	40.1	3	
17765	peflin	PEF1	ER-to-Golgi transport, calcium-dependent adaptor, collagen export	insertion	G14-Gal4X GD8610	v32404	+	0.5	15	30.2	8	McGourty et al., 2016[6]
							-	1.1	35	32.4	12	

Supplementary Table 2: Absolute values for normalized data and additional statistical details. The figure and panel, genotype, and conditions are noted. Average values (with standard error values noted in parentheses) are shown for all data. For electrophysiological experiments, passive membrane properties (input resistance, leak current), mEPSC, EPSC, quantal content (QC), data samples (n), and statistical significance tests and values are shown.

Figure	Label	Genotype	PhTx	mEPSC amplitude (nA)	EPSC amplitude (nA)	QC	mEPSC frequency (Hz)	Input resistance (MΩ)	Leak current (nA)	n	P Value (significance: mEPSC amp, EPSC, QC, mEPSC freq)
2c,d	wild type	w^{1118}	-	0.416 (0.010)	47.539 (6.205)	116.894 (15.715)	2.538 (0.297)	19.000 (2.154)	4.403 (0.483)	17	-
2c,d	wild type	w^{1118}	+	0.190 (0.014)	40.084 (6.864)	209.751 (30.411)	1.818 (0.100)	11.364 (0.877)	4.982 (0.669)	11	<0.0001 (****), 0.4359 (ns), 0.0062 (**), 0.0699 (ns)
2c,d	inc^{kk3}	inc^{kk3}	-	0.377 (0.019)	40.591 (3.403)	109.991 (10.179)	1.765 (0.216)	14.188 (2.801)	3.930 (0.532)	16	-
2c,d	inc^{kk3}	inc^{kk3}	+	0.203 (0.014)	26.661 (4.994)	128.007 (20.924)	0.876 (0.159)	23.700 (3.113)	1.506 (0.290)	10	<0.0001 (****), 0.0251 (*), 0.3963 (ns), 0.0072 (**)
2d	$inc^{kk3/Df}$	inc^{kk3}/inc^{Df}	-	0.327 (0.009)	42.173 (6.412)	129.429 (20.323)	1.750 (0.159)	6.900 (1.140)	1.236 (0.319)	10	-
2d	$inc^{kk3/Df}$	inc^{kk3}/inc^{Df}	+	0.143 (0.007)	13.508 (2.437)	98.420 (21.895)	0.478 (0.064)	5.375 (0.263)	3.603 (0.415)	8	<0.0001 (****), 0.0016 (**), 0.3167 (ns), <0.0001 (****)
2d	inc^{kk4}	inc^{kk4}	-	0.475 (0.013)	47.175 (4.913)	99.888 (10.597)	1.033 (0.119)	6.929 (0.529)	2.165 (0.263)	14	-
2d	inc^{kk4}	inc^{kk4}	+	0.194 (0.009)	20.077 (2.272)	108.39 (14.284)	1.689 (0.223)	5.857 (0.275)	3.148 (0.264)	14	<0.0001 (****), <0.0001 (****), 0.6366 (ns), <0.0153 (*)
2g	wild type	w^{1118}	-	0.439 (0.015)	42.118 (5.837)	98.757 (15.023)	2.372 (0.412)	9.300 (1.777)	3.730 (0.650)	10	
2g	wild type	w^{1118}	+	0.207 (0.014)	37.289 (3.304)	180.545 (11.241)	1.421 (0.311)	10.444 (1.590)	3.139 (0.683)	9	<0.0001 (****), 0.4992 (ns), 0.0006 (***), 0.0022 (*)
2f,g	presynaptic rescue	inc^{kk3}; OK371-Gal4/ UAS-smFP-Inc	-	0.342 (0.021)	41.817 (2.837)	130.907 (13.521)	2.063 (0.279)	7.125 (0.730)	2.321 (0.486)	16	-
2f,g	presynaptic rescue	inc^{kk3}; OK371-Gal4/UAS-smFP-Inc	+	0.163 (0.011)	22.532 (2.420)	148.699 (18.871)	0.884 (0.129)	5.714 (0.450)	3.051 (0.211)	14	<0.0001 (****), <0.0001 (****), 0.4418 (ns), <0.0010 (**)
2f,g	postsynaptic rescue	inc^{kk3}; UAS-smFP-Inc/+; MHC-Gal4/+	-	0.321 (0.023)	46.367 (6.799)	149.643 (34.958)	3.410 (0.112)	5.750 (0.305)	2.188 (0.238)	12	-
2f,g	postsynaptic rescue	inc^{kk3}; UAS-smFP-Inc/+; MHC-Gal4/+	+	0.178 (0.010)	42.524 (4.441)	249.018 (29.998)	1.669 (0.291)	5.833 (0.405)	3.342 (0.306)	10	<0.0001 (****), 0.5096 (ns), 0.0071 (**), 0.0137 (*)
				QC % baseline significance: postsynaptic rescue +PhTx v.s. wild type + PhTx: 0.8811 (ns)							
3a,b	pre>Cul3 RNAi	OK371-Gal4/UAS-Cul3 $RNAi^{11861R}$; UAS-Dcr2/+	-	0.342 (0.015)	36.546 (3.014)	107.969 (9.333)	2.363 (0.303)	7.500 (0.732)	1.275 (0.434)	8	-
3a,b	pre>Cul3 RNAi	OK371-Gal4/UAS-Cul3 $RNAi^{11861R}$; UAS-Dcr2/+	+	0.176 (0.012)	29.324 (2.873)	165.346 (7.621)	1.144 (0.164)	8.000 (0.906)	2.210 (0.325)	8	<0.0001 (****), 0.1048 (ns), 0.0003 (***), 0.0033 (**)
3a,b	post> Cul3 RNAi	UAS-Cul3 $RNAi^{11861R}$/+; MHC-Gal4/UAS-Dcr2	-	0.362 (0.012)	44.023 (4.485)	127.653 (16.999)	3.603 (0.696)	6.231 (0.257)	2.082 (0.361)	13	-

3a,b	post> Cul3 RNAi	*UAS-Cul3RNAi*^{11861R}*/+; MHC-Gal4/UAS-Dcr2*	+	0.206 (0.012)	23.343 (2.183)	119.502 (13.153)	1.557 (0.153)	6.500 (0.294)	2.764 (0.373)	18	<0.0001 (****), 0.0001 (***), 0.7029 (ns), 0.0024 (**)
3c,d	*inc*^{kk3}*/+*	*inc*^{kk3}*/+*	-	0.389 (0.009)	47.674 (3.309)	124.351 (10.291)	2.908 (0.365)	8.182 (0.519)	2.160 (0.288)	11	-
3c,d	*inc*^{kk3}*/+*	*inc*^{kk3}*/+*	+	0.208 (0.011)	48.392 (4.610)	235.243 (20.197)	1.783 (0.318)	7.909 (0.653)	2.734 (0.232)	11	<0.0001 (****), 0.9006 (ns), <0.0001 (****), 0.0309 (*)
3c,d	*Cul3*^{EY11031}*/+*	*Cul3*^{EY11031}*/+*	-	0.346 (0.015)	30.569 (2.645)	88.891 (7.429)	2.629 (0.654)	7.000 (0.732)	2.109 (0.488)	8	-
3c,d	*Cul3*^{EY11031}*/+*	*Cul3*^{EY11031}*/+*	+	0.182 (0.019)	26.283 (2.029)	158.581 (21.750)	1.288 (0.321)	6.875 (0.673)	2.896 (0.689)	8	<0.0001 (****), 0.2194 (ns), 0.0090 (**), 0.0868 (*)
3c,d	*inc*^{kk3}*/+; Cul3*^{EY11031}*/+*	*inc*^{kk3}*/+; Cul3*^{EY11031}*/+*	-	0.350 (0.015)	51.326 (3.331)	150.141 (12.620)	2.041 (0.141)	6.545 (0.493)	3.074 (0.761)	11	-
3c,d	*inc*^{kk3}*/+; Cul3*^{EY11031}*/+*	*inc*^{kk3}*/+; Cul3*^{EY11031}*/+*	+	0.181 (0.016)	22.134 (2.622)	140.291 (30.912)	1.522 (0.146)	5.571 (0.291)	3.199 (0.184)	14	<0.0001 (****), <0.0001 (****), 0.7911 (*), 0.0199 (*)
6d,e	wild type	*w*^{1118}	-	0.447 (0.012)	47.565 (2.473)	108.186 (7.942)	2.746 (0.165)	7.417 (0.557)	2.461 (0.314)	8	-
6d,e	wild type	*w*^{1118}	+	0.249 (0.004)	44.050 (2.002)	176.555 (7.064)	1.492 (0.210)	6.714 (0.484)	2.978 (0.402)	7	<0.0001 (****), 0.1773 (ns), 0.0005 (***), 0.0008 (***)
6d,e	*pef*^{C295}	*pef*^{C295}	-	0.439 (0.006)	41.957 (7.354)	96.183 (17.799)	3.709 (0.731)	5.571 (0.686)	1.391 (0.226)	7	-
6d,e	*pef*^{C295}	*pef*^{C295}	+	0.261 (0.004)	17.920 (2.936)	68.404 (10.666)	2.106 (0.222)	6.750 (0.407)	1.515 (0.113)	8	<0.0001 (****), 0.0041 (**), 0.2896 (ns), 0.1240 (ns)
6d,e	*muscle*>Pef RNAi	*G14-Gal4/UAS-Pef RNAi*	-	0.399 (0.011)	34.753 (2.457)	89.116 (8.176)	2.342 (0.233)	7.154 (0.406)	3.817 (0.500)	10	-
6d,e	*muscle*>Pef RNAi	*G14-Gal4/UAS-Pef RNAi*	+	0.202 (0.007)	12.856 (1.610)	63.940 (8.205)	0.760 (0.065)	6.000 (0.677)	3.904 (0.292)	9	<0.0001 (****), <0.0001 (****), 0.09602 (ns), <0.0001 (****)
6f,g	*pef*^{C295}*/+*	*pef*^{C295}*/+*	-	0.435 (0.011)	44.373 (0.932)	102.338 (3.180)	2.684 (0.231)	7.000 (0.559)	1.846 (0.234)	8	-
6f,g	*pef*^{C295}*/+*	*pef*^{C295}*/+*	+	0.230 (0.007)	44.771 (3.859)	192.562 (12.384)	1.126 (0.073)	6.625 (0.323)	2.688 (0.284)	8	<0.0001 (****), 0.9156 (ns), <0.0001 (****), 0.0312 (*)
6f,g	*Cul3*^{EY11031}*/+*	*Cul3*^{EY11031}*/+*	-	0.436 (0.023)	35.969 (4.768)	80.761 (7.756)	2.578 (0.678)	8.750 (0.861)	2.673 (0.465)	8	-
6f,g	*Cul3*^{EY11031}*/+*	*Cul3*^{EY11031}*/+*	+	0.260 (0.012)	38.819 (2.648)	149.363 (8.392)	1.183 (0.065)	6.000 (0.436)	2.773 (0.289)	7	<0.0001 (****), 0.6245 (ns), <0.0001 (****), 0.0784 (ns)
6f,g	*Cul3*^{EY11031}*/+, pef*^{C295}*/+*	*Cul3*^{EY11031}*/+, pef*^{C295}*/+*	-	0.440 (0.015)	35.924 (1.752)	83.821 (4.780)	2.005 (0.167)	7.000 (0.281)	2.347 (0.319)	21	-
6f,g	*Cul3*^{EY11031}*/+, pef*^{C295}*/+*	*Cul3*^{EY11031}*/+, pef*^{C295}*/+*	+	0.239 (0.009)	20.925 (2.825)	85.701 (9.919)	1.574 (0.567)	6.750 (0.579)	2.503 (0.195)	11	<0.0001 (****), <0.0001 (****), 0.854 (ns), 0.383 (ns)
6h,i	*inc*^{kk3}*/+*	*inc*^{kk3}*/+*	-	0.391 (0.010)	47.143 (4.321)	122.094 (13.060)	2.645 (0.373)	8.375 (0.653)	2.135 (0.385)	8	-
6h,i	*inc*^{kk3}*/+*	*inc*^{kk3}*/+*	+	0.207 (0.014)	46.001 (5.278)	225.273 (24.012)	1.893 (0.398)	7.889 (0.837)	2.821 (0.273)	8	<0.0001 (****), 0.1007 (ns), 0.0011 (**), 0.1007 (ns)

6h,i	*dmp*^{253}/+	*dmp*^{253}/+	-	0.457 (0.013)	41.966 (2.637)	89.099 (6.356)	1.844 (0.191)	8.938 (1.206)	1.218 (0.116)	8	-
6h,i	*dmp*^{253}/+	*dmp*^{253}/+	+	0.206 (0.008)	35.423 (2.265)	173.986 (10.497)	0.877 (0.067)	8.222 (0.440)	1.527 (0.605)	14	<0.0001 (****),<br>0.1183 (ns),<br>0.0001 (***),<br>0.0003 (***)
6h,i	*inc*^{kk3}/+;<br>*dmp*^{253}/+	*inc*^{kk3}/+; *dmp*^{253}/+	-	0.368 (0.011)	32.367 (2.285)	87.627 (4.685)	1.688 (0.209)	5.778 (0.324)	1.510 (0.378)	9	-
6h,i	*inc*^{kk3}/+;<br>*dmp*^{253}/+	*inc*^{kk3}/+; *dmp*^{253}/+	+	0.180 (0.007)	16.738 (1.691)	94.482 (11.231)	0.836 (0.069)	6.111 (0.389)	1.177 (0.385)	9	<0.0001 (****),<br>0.0035 (**),<br>0.686 (ns),<br>0.0202 (*)
6h,i	*Cul3*^{EY11031}/+;<br>*dmp*^{253}/+	*Cul3*^{EY11031}/+;<br>*dmp*^{253}/+	-	0.420 (0.014)	30.724 (1.426)	73.423 (2.824)	1.527 (0.145)	5.667 (0.303)	1.453 (0.358)	10	-
6h,i	*Cul3*^{EY11031}/+;<br>*dmp*^{253}/+	*Cul3*^{EY11031}/+;<br>*dmp*^{253}/+	+	0.232 (0.010)	11.697 (1.364)	50.456 (5.379)	0.584 (0.087)	7.462 (0.584)	1.748 (0.385)	11	<0.0001 (****),<br><0.0001 (****),<br>0.0038 (**),<br>0.0001 (***)
S1b-f	*wild type*	*w1118*	-	0.416 (0.011)	47.539 (6.205)	116.894 (15.715)	2.538 (0.297)	9.000 (2.154)	4.403 (0.483)	17	-
S1b-f	*inckk3*	*inckk3*	-	0.377 (0.019)	40.591 (3.403)	109.991 (10.179)	1.765 (0.216)	14.188 (2.801)	3.930 (0.532)	16	0.0797 (ns),<br>0.3422 (ns),<br>0.7185 (ns),<br>0.0460 (ns)
S1b-f	*inc2*	*inc2*	-	0.416 (0.013)	42.522 (4.934)	101.760 (11.103)	0.814 (0.198)	10.143 (0.705)	3.516 (0.389)	7	0.9848 (ns),<br>0.6299 (ns),<br>0.5628 (ns),<br>0.0018 (**)
S1b-f	*inc2/Df*	*inc2/incDf*	-	0.394 (0.012)	41.214 (5.634)	103.545 (13.177)	1.599 (0.270)	6.667 (0.376)	2.796 (0.450)	12	0.1840 (ns),<br>0.4778 (ns),<br>0.5451 (ns),<br>0.0474 (ns)
S1b-f	*inc2/1*	*inc2/inc1*	-	0.365 (0.009)	25.988 (2.730)	72.830 (8.496)	0.987 (0.167)	8.286 (0.997)	3.534 (0.635)	14	0.0015 (**),<br>0.0061 (**),<br>0.0276 (*),<br>0.0002 (***)
S2c,d	-	*w*^{1118}	-	0.416 (0.010)	47.539 (6.205)	116.894 (15.715)	2.538 (0.297)	19.000 (2.154)	4.403 (0.483)	17	-
S2c,d	*GluRIIA*	*GluRIIA*^{SP16}	-	0.175 (0.009)	33.330 (2.351)	193.522 (11.376)	0.758 (0.231)	11.700 (0.684)	3.712 (0.250)	10	<0.0001 (****),<br>0.1084 (ns),<br>0.0037 (**),<br>0.0007 (***)
S2c,d	*inc*^{kk3},<br>*GluRIIA*	*inc*^{kk3};<br>*GluRIIA*^{SP16}	+	0.202 (0.011)	20.0129 (3.003)	106.939 (19.961)	0.923 (0.547)	4.700 (0.456)	3.530 (0.471)	10	<0.0001 (****),<br>0.0038 (**),<br>0.7225 (ns),<br>0.0183 (*)
S2c,d	inc$^{kk3/Df}$,<br>*GluRIIA*	*inc*^{kk3}/*inc*^{Df};<br>*GluRIIA*^{SP16}	+	0.180 (0.007)	21.987 (2.984)	118.852 (13.807)	1.610 (0.359)	6.000 (0.365)	2.028 (0.393)	15	<0.0001 (****),<br>0.0013 (**),<br>0.9269 (ns),<br>0.0540 (ns)
S3a-d	wild type	*w*^{1118}	-	0.419 (0.010)	47.656 (5.194)	114.300 (12.286)	2.869 (0.335)	8.125 (0.706)	2.686 (0.312)	16	-
S3a-d	neuronal ><br>*smFP-inc*	*OK371-Gal4/UAS-smFP-inc*	-	0.387 (0.007)	44.644 (4.622)	116.406 (12.768)	1.633 (0.275)	5.666 (0.337)	1.917 (0.546)	9	0.0377 (ns),<br>0.7080 (ns),<br>0.9151 (ns),<br>0.0222 (*)
not shown	neuronal ><br>*smFP-inc*	*OK371-Gal4/UAS-smFP-inc*	+	0.211 (0.006)	43.651 (4.304)	206.877 (20.398)	0.914 (0.388)	6.235 (0.561)	2.328 (0.432)	8	<0.0001 (****),<br>0.8781 (ns),<br><0.0001 (****),<br>0.1448 (ns)
S3a-d	muscle ><br>*smFP-inc*	*MHC-Gal4/UAS-smFP-inc*	-	0.397 (0.016)	41.769 (2.760)	107.062 (7.949)	3.610 (0.597)	6.929 (0.486)	2.643 (0.363)	14	0.2380 (ns),<br>0.3453 (ns),<br>0.6356 (ns),<br>0.2727 (ns)
not shown	muscle ><br>*smFP-inc*	*MHC-Gal4/UAS-smFP-inc*	+	0.199 (0.009)	40.532 (3.983)	199.679 (20.015)	0.751 (0.286)	6.644 (0.354)	3.776 (0.648)	9	<0.0001 (****),<br>0.7945 (ns),<br><0.0001 (****),<br>0.0015 (**)

S3e,f	wild type	w^{1118}	-	0.423 (0.011)	44.171 (3.312)	106.268 (9.882)	2.413 (0.263)	8.100 (0.482)	2.734 (0.905)	10	-
S3e,f	wild type	w^{1118}	+	0.179 (0.006)	37.310 (2.348)	210.0126 (14.121)	0.871 (0.101)	7.600 (0.542)	2.736 (0.515)	10	<0.0001 (****), 0.1082 (ns), <0.0001 (****), <0.0001 (****)
S3e,f	inc^{smFP}	inc^{smFP}	-	0.380 (0.013)	40.186 (4.975)	108.034 (14.523)	0.914 (0.123)	5.818 (0.296)	2.345 (0.352)	11	-
S3e,f	inc^{smFP}	inc^{smFP}	+	0.238 (0.014)	48.286 (4.277)	206.972 (19.818)	1.050 (0.176)	5.900 (0.458)	2.917 (0.449)	10	<0.0001 (****), 0.2365 (ns), 0.0006 (***), 0.5271 (ns)

Figure	Label	Genotype	PhTx	pCaMKII intensity (% wild type)	BRP intensity (% wild type)	n (pCaMKII, BRP)	P value (significance: pCaMKII, BRP)
3e,f	wild type	w^{1118}	-	100.000 (4.585)	100.000 (9.194)	27, 10	-
3e,f	wild type	w^{1118}	+	38.220 (3.565)	149.283 (6.613)	14, 11	<0.0001 (****), 0.0003 (***)
3e,f	inc^{kk3}	inc^{kk3}	-	115.120 (8.656)	96.930 (10.672)	16, 15	0.1930 (ns), 0.8408 (ns)
3e,f	inc^{kk3}	inc^{kk3}	+	53.920 (3.883)	101.273 (13.716)	19, 10	<0.0001 (****), 0.9394 (ns)
				pCaMKII significance:inc^{kk3}+PhTx v.s. wild type+PhTx: 0.2433 (ns)			
not shown	inc^{kk4}	inc^{kk4}	-	113.300 (5.848)	100.253 (6.882)	17, 11	0.3953 (ns), 0.9819 (ns)
not shown	inc^{kk4}	inc^{kk4}	+	55.940 (3.1777)	94.549 (14.093)	18, 10	<0.0001 (****), 0.7399 (ns)

Figure	Label	Genotype	PhTx	Inc^{smFP} intensity – HRP+DLG area (% baseline total NMJ signal)	Inc^{smFP} intensity – Dlg only area (% baseline total NMJ signal)	n	P value (significance)
4a,c	inc^{smFP}	inc^{smFP}	-	77.125 (6.566)	22.875 (4.871)	21	-
4a,c	inc^{smFP}	inc^{smFP}	+	113.730 (8.221)	53.344 (5.757)	15	HRP: 0.0013 (**) Dlg-HRP: 0.0003 (***)

Figure	Label	Genotype	PhTx	cytosolic Inc^{smFP} intensity (% baseline)	n	P value (significance)
not shown	inc^{smFP}	inc^{smFP}	-	100.000 (5.647)	9	-
not shown	inc^{smFP}	inc^{smFP}	+	96.762 (9.371)	9	0.7711 (ns)

Figure	Label	Genotype	PhTx	Inc^{smFP} intensity – HRP+DLG area (% baseline)	Inc^{smFP} intensity – Dlg only area (% baseline)	n	P value (significance)
4d,e	presynaptic>Inc RNAi	inc^{smFP};*OK6-Gal4/UAS-Inc RNAi*	-	100.000 (11.220)	100.000 (16.220)	10	-
4d,e	presynaptic>Inc RNAi	inc^{smFP};*OK6-Gal4/UAS-Inc RNAi*	+	173.322 (17.114)	177.493 (30.360)	11	HRP: 0.0024 (**) Dlg-HRP: 0.0415 (*)
not shown	presynaptic> Inc RNAi	inc^{smFP};*OK6-Gal4/UAS-Inc RNAi*	-	47.945 (5.379)	83.204 (13.496)	10	HRP: 0.0004 (***) Dlg-HRP: 0.6087 (ns)
not shown	postsynaptic> Inc RNAi	inc^{smFP};*G14-Gal4/UAS-Inc RNAi*	-	113.335 (12.553)	44.066 (7.378)	11	HRP: 0.391 (ns) Dlg-HRP: 0.0003 (***)

Figure	Label	Genotype	PhTx	Cul3-Flag intensity (%baseline)	n	P value (significance)
4f,g	muscle>*Flag-Cul3*	*G14-Gal4/UAS-3xHA-3xFlag-Cul3*	-	100.000 (2.065)	19	-
4f,g	muscle>*Flag-Cul3*	*G14-Gal4/UAS-3xHA-3xFlag-Cul3*	+	128.605 (2.775)	18	<0.0001 (****)

Figure	Label	Genotype	PhTx	αFK2 ubiquitin intensity (% wild type)	n	P value (significance)
5a,c	wild type	w^{1118}	-	100.000 (6.351)	10	-
5a,c	wild type	w^{1118}	+	138.638 (7.950)	10	0.0017 (**)
5a,c	inc^{kk3}	inc^{kk3}	-	112.068 (5.885)	10	0.6010 (ns)
5a,c	inc^{kk3}	inc^{kk3}	+	102.165 (6.983)	10	0.9960 (ns)
		αFK2 significance:inc^{kk3}+PhTx v.s. inc^{kk3}: 0.7364 (ns)				

Figure	Label	Genotype	PhTx	αFK1 ubiquitin intensity (%wild type)	n	P value (significance)

5a,d	wild type	w^{1118}	-	100.000 (8.422)	17	-
5a,d	wild type	w^{1118}	+	116.907 (11.569)	14	0.6677 (ns)
5a,d	inc^{kk3}	inc^{kk3}	-	105.744 (9.796)	21	0.9736 (ns)
5a,d	inc^{kk3}	inc^{kk3}	+	121.628 (8.672)	22	0.3707 (ns)
		αFK1 significance:inc^{kk3}+PhTx v.s. inc^{kk3}: 0.5885 (ns)				

**Figure**	**Label**	**Genotype**	**PhTx**	**αFK2 ubiquitin intensity (% wild type)**	**n**	**P value (significance)**
5e,f	wild type	w^{1118}	-	100.000 (8.335)	16	
not shown	wild type	w^{1118}	+	148.473 (17.647)	13	0.0136 (*)
5e,f	presynaptic rescue	$inc^{kk3/y}$;OK371-Gal4/UAS-smFP-Inc	-	111.553 (13.162)	10	0.4421 (ns)
5e,f	presynaptic rescue	$inc^{kk3/y}$;OK371-Gal4/UAS-smFP-Inc	+	129.665 (15.374)	10	0.3867 (ns)
5e,f	postsynaptic rescue	$inc^{kk3/y}$;G14-Gal4/UAS-smFP-Inc	-	85.138 (9.186)	12	0.2448 (ns)
5e,f	postsynaptic rescue	$inc^{kk3/y}$;G14-Gal4/UAS-smFP-Inc	+	147.708 (20.738)	11	0.0098 (**)

**Figure**	**Label**	**Genotype**	**Total daily sleep (min)**	**n**	**P value (significance)**
S1g	wild type	w^{1118}	731.18 (25.15)	30	-
S1g	$inc^{2/1}$	inc^{2}/inc^{1}	501.98 (38.94)	28	p<0.0001 (****)
S1g	$inc^{kk3/1}$	inc^{kk3}/inc^{1}	418.63 (35.93)	25	p<0.0001 (****)
S1g	$inc^{kk4/1}$	inc^{kk4}/inc^{1}	334.18 (63.61)	10	p<0.0001 (****)

**Figure**	**Label**	**Genotype**	**Total daily sleep (min)**	**n**	**P value (significance)**
S1h	wild type	w^{1118}	996.16 (20.62)	27	-
S1h	UAS-smFP-Inc/+	UAS-smFP-Inc/+	934.51 (20.75)	28	ns
S1h	inc^{1}, inc-Gal4/+	inc^{1}, inc-Gal4/+	480.94 (31.69)	16	p<0.0001 (****)
S1h	inc^{1}, inc-Gal4/UAS-smFP-Inc	inc^{1}, inc-Gal4/UAS-smFP-Inc	952.90 (16.52)	43	ns

**Figure**	**Label**	**Genotype**	**PhTx**	**GluRIIA intensity (% wild type)**	**GluRIID intensity (% wild type)**	**n**	**P value (significance: GluRIIA, GluRIID)**
S2a,b	wild type	w^{1118}	-	100.000 (5.262)	100.000 (3.948)	10	-
S2a,b	inc^{kk3}	inc^{kk3}	-	93.777 (6.088)	103.001 (5.910)	13	0.4643 (ns), 0.6968 (ns)
not shown	inc^{kk4}	inc^{kk4}	-	100.528 (7.178)	111.965 (7.701)	15	0.9576 (ns), 0.2446 (ns)

Supplementary References

1 Bennett, E. J., Rush, J., Gygi, S. P. & Harper, J. W. Dynamics of cullin-RING ubiquitin ligase network revealed by systematic quantitative proteomics. Cell 143, 951-965 (2010).

2 Giot, L. et al. A protein interaction map of Drosophila melanogaster. Science 302, 1727-1736 (2003).

3 Hein, M. Y. et al. A human interactome in three quantitative dimensions organized by stoichiometries and abundances. Cell 163, 712-723 (2015).

4 Hudson, A. M., Mannix, K. M. & Cooley, L. Actin Cytoskeletal Organization in Drosophila Germline Ring Canals Depends on Kelch Function in a Cullin-RING E3 Ligase. Genetics 201, 1117-1131 (2015).

5 Hutchins, J. R. et al. Systematic analysis of human protein complexes identifies chromosome segregation proteins. Science 328, 593-599 (2010).

6 McGourty, C. A. et al. Regulation of the CUL3 Ubiquitin Ligase by a Calcium-Dependent Co-adaptor. Cell 167, 525-538 e514 (2016).

7 Wang, J. et al. Toward an understanding of the protein interaction network of the human liver. Mol Syst Biol 7, 536 (2011).
