# Supplementary material for: Cul3 and insomniac are required for rapid ubiquitination of postsynaptic targets and retrograde homeostatic signaling
Source: Nat Commun. 2019 Jul 5;10:2998. doi: 10.1038/s41467-019-10992-6 (PMC6611771; doi:10.1038/s41467-019-10992-6)
Supplement: Supplementary file 3 — Description of Additional Supplementary Files [file 41467_2019_10992_MOESM3_ESM.pdf]

Description of Additional Supplementary Files

File Name: Supplementary Data 1
Description: List of genes screened and summarized results. The gene identity (noted by CG number), gene name, putative function, genotype, source, genetic perturbation, PhTx application and mEPSP, EPSP, and quantal content values are shown for each gene screened.
