# Supplementary material for: Cul3 and insomniac are required for rapid ubiquitination of postsynaptic targets and retrograde homeostatic signaling
Source: Nat Commun. 2019 Jul 5;10:2998. doi: 10.1038/s41467-019-10992-6 (PMC6611771; doi:10.1038/s41467-019-10992-6)
Supplement: Supplementary file 5 — Reporting Summary [file 41467_2019_10992_MOESM5_ESM.pdf]

nature research

Corresponding author(s): Dion Dickman

Reporting Summary

Nature Research wishes to improve the reproducibility of the work that we publish. This form provides structure for consistency and transparency in reporting. For further information on Nature Research policies, see Authors & Referees and the Editorial Policy Checklist.

Statistical parameters

When statistical analyses are reported, confirm that the following items are present in the relevant location (e.g. figure legend, table legend, main text, or Methods section).

n/a	Confirmed	
☐	☒	The exact sample size (n) for each experimental group/condition, given as a discrete number and unit of measurement
☐	☒	An indication of whether measurements were taken from distinct samples or whether the same sample was measured repeatedly
☐	☒	The statistical test(s) used AND whether they are one- or two-sided<br>*Only common tests should be described solely by name; describe more complex techniques in the Methods section.*
☐	☒	A description of all covariates tested
☐	☒	A description of any assumptions or corrections, such as tests of normality and adjustment for multiple comparisons
☐	☒	A full description of the statistics including central tendency (e.g. means) or other basic estimates (e.g. regression coefficient) AND variation (e.g. standard deviation) or associated estimates of uncertainty (e.g. confidence intervals)
☐	☒	For null hypothesis testing, the test statistic (e.g. F, t, r) with confidence intervals, effect sizes, degrees of freedom and P value noted<br>*Give P values as exact values whenever suitable.*
☒	☐	For Bayesian analysis, information on the choice of priors and Markov chain Monte Carlo settings
☐	☒	For hierarchical and complex designs, identification of the appropriate level for tests and full reporting of outcomes
☒	☐	Estimates of effect sizes (e.g. Cohen's d, Pearson's r), indicating how they were calculated
☐	☒	Clearly defined error bars<br>*State explicitly what error bars represent (e.g. SD, SE, CI)*

Our web collection on statistics for biologists may be useful.

Software and code

Policy information about availability of computer code

Data collection	Clampex 10.7 (Molecular Divices), Axoclamp (Molecular Divices), NIS Elements software
Data analysis	Clampfit 10.7 (Molecular Divices), MiniAnalysis (Synaptosoft), Excel (Microsoft), GraphPad Prism (GraphPad Software), NIS Elements software General Analysis toolkit

For manuscripts utilizing custom algorithms or software that are central to the research but not yet described in published literature, software must be made available to editors/reviewers upon request. We strongly encourage code deposition in a community repository (e.g. GitHub). See the Nature Research guidelines for submitting code & software for further information.

Data

Policy information about availability of data

All manuscripts must include a data availability statement. This statement should provide the following information, where applicable:

- Accession codes, unique identifiers, or web links for publicly available datasets
- A list of figures that have associated raw data
- A description of any restrictions on data availability

Data that support the findings of this study are available within the manuscript and includes a variety of additional details in the supplementary information and data source files.

Field-specific reporting

Please select the best fit for your research. If you are not sure, read the appropriate sections before making your selection.

☒ Life sciences ☐ Behavioural & social sciences ☐ Ecological, evolutionary & environmental sciences

For a reference copy of the document with all sections, see nature.com/authors/policies/ReportingSummary-flat.pdf

Life sciences study design

All studies must disclose on these points even when the disclosure is negative.

Sample size	This information can be found in the figure legends, data source file, and supplemental tables.
Data exclusions	This information can be found in the Methods section.
Replication	The information of reproducibility of experiments in this paper can be found in the materials and methods section and supplemental tables.
Randomization	This information can be found in the Methods and supplemental tables.
Blinding	This information can be found in the Methods.

Reporting for specific materials, systems and methods

Materials & experimental systems

n/a	Involved in the study	
☐	☒	Unique biological materials
☐	☒	Antibodies
☒	☐	Eukaryotic cell lines
☒	☐	Palaeontology
☐	☒	Animals and other organisms
☒	☐	Human research participants

Methods

n/a	Involved in the study	
☒	☐	ChIP-seq
☒	☐	Flow cytometry
☒	☐	MRI-based neuroimaging

Unique biological materials

Policy information about availability of materials

Obtaining unique materials	All unique materials are available by request or are available from commercial vendors. Should this manuscript be published, we will donate unique Drosophila stocks to the Bloomington Drosophila Stock Center, as we have with other unique stocks generated.

Antibodies

Antibodies used	Details provided in the Methods section in the manuscript.
Validation	Details provided in the manuscript.

Animals and other organisms

Policy information about studies involving animals; ARRIVE guidelines recommended for reporting animal research

Laboratory animals	Details provided in the Methods section in the manuscript.
Wild animals	This study did not involve wild animals.
Field-collected samples	This study did not involve animals collected from the field.
